# Diverse and reprogrammable mechanisms of malignant cell transformation in lymphocytes: pathogenetic insights and translational implications

**DOI:** 10.3389/fonc.2024.1383741

**Published:** 2024-04-03

**Authors:** Mariusz A. Wasik, Patricia M. Kim, Reza Nejati

**Affiliations:** ^1^ Department of Pathology, Fox Chase Cancer Center, Philadelphia, PA, United States; ^2^ Department of Pathology and Laboratory Medicine, Penn State College of Medicine, Hershey, PA, United States

**Keywords:** B-cell lymphoma, T-cell lymphoma, pathogenic signaling plasticity, dedifferentiation, transdifferentiation

## Abstract

While normal B- and T-lymphocytes require antigenic ligands to become activated via their B- and T-cell receptors (BCR and TCR, respectively), B- and T-cell lymphomas show the broad spectrum of cell activation mechanisms regarding their dependence on BCR or TCR signaling, including loss of such dependence. These mechanisms are generally better understood and characterized for B-cell than for T-cell lymphomas. While some lymphomas, particularly the indolent, low-grade ones remain antigen-driven, other retain dependence on activation of their antigen receptors seemingly in an antigen-independent manner with activating mutations of the receptors playing a role. A large group of lymphomas, however, displays complete antigen receptor independence, which can develop gradually, in a stepwise manner or abruptly, through involvement of powerful oncogenes. Whereas some of the lymphomas undergo activating mutations of genes encoding proteins involved in signaling cascades downstream of the antigen-receptors, others employ activation mechanisms capable of substituting for these BCR- or TCR-dependent signaling pathways, including reliance on signaling pathways physiologically activated by cytokines. Finally, lymphomas can develop cell-lineage infidelity and in the extreme cases drastically rewire their cell activation mechanisms and engage receptors and signaling pathways physiologically active in hematopoietic stem cells or non-lymphoid cells. Such profound reprograming may involve partial cell dedifferentiation or transdifferentiation towards histocytes, dendritic, or mesodermal cells with various degree of cell maturation along these lineages. In this review, we elaborate on these diverse pathogenic mechanisms underlying cell plasticity and signaling reprogramming as well as discuss the related diagnostic and therapeutic implications and challenges.

## Introduction

An unprecedented progress in molecular technologies enabled broad and in-depth studies of normal and malignant cells and tissues. These studies provided us with detailed insights into the mechanisms of carcinogenesis, permitting novel approaches to management of patients with the whole spectrum of malignancies, with lymphomas being a prime example. It also revealed considerable diversity of oncogenic mechanisms as well as a remarkable ability of malignant cells to reprogram intracellular signaling pathways to sustain their survival and proliferation. An in-depth understanding of pathogenic mechanisms activating and sustaining transformed cells at any given stage of lymphoid malignancy, including recognition of their plasticity resulting in reprogramming cell-signaling dependency during lymphoma progression or at its recurrence, should lead to precisely targeted and highly individualized therapies.

## Pathogenic role of antigen-dependent activation of B-cell receptors and T-cell receptors in lymphomas

**Figure 1:**
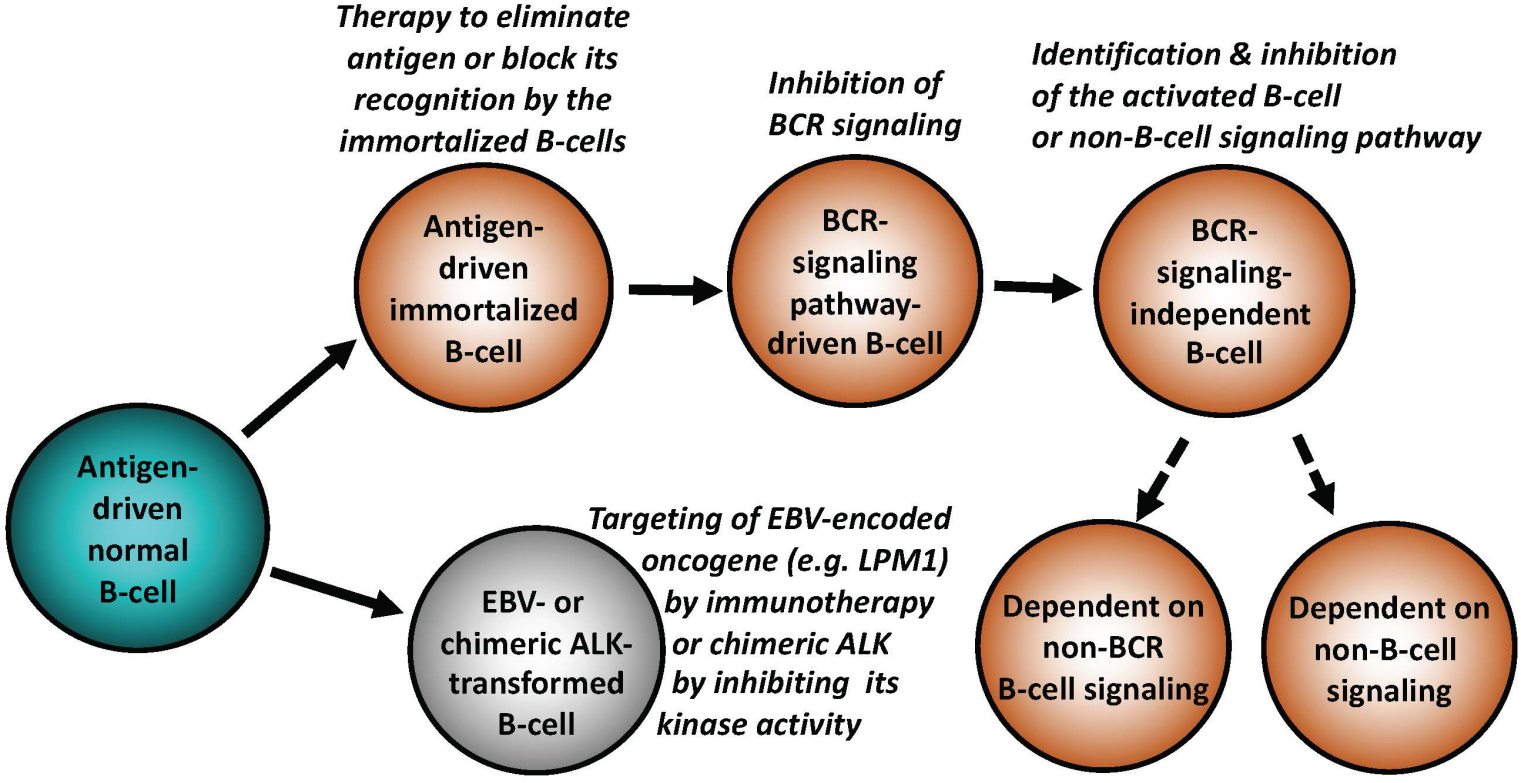
Key mechanisms of malignant cell transformation in B-cell lymphomas. While indolent (“low-grade”) B-cell lymphomas frequently remain antigen dependent, more advanced lymphomas relay on BCR in the antigen-independent manner, signaling pathways down-stream of BCR, non-BCR signaling pathways physiologically activated in normal mature or immature B lymphocytes, or, in extreme cases, develop reliance on signaling pathways normally activated in non-lymphoid types of mesenchymal cells. EBV and chimeric ALK (typically CLTC::ALK for B cells) are able to potently transform normal B lymphocytes. Types of targeted therapies may need to be adjusted to the specific B cell-transforming mechanisms, as listed.

**Figure 2:**
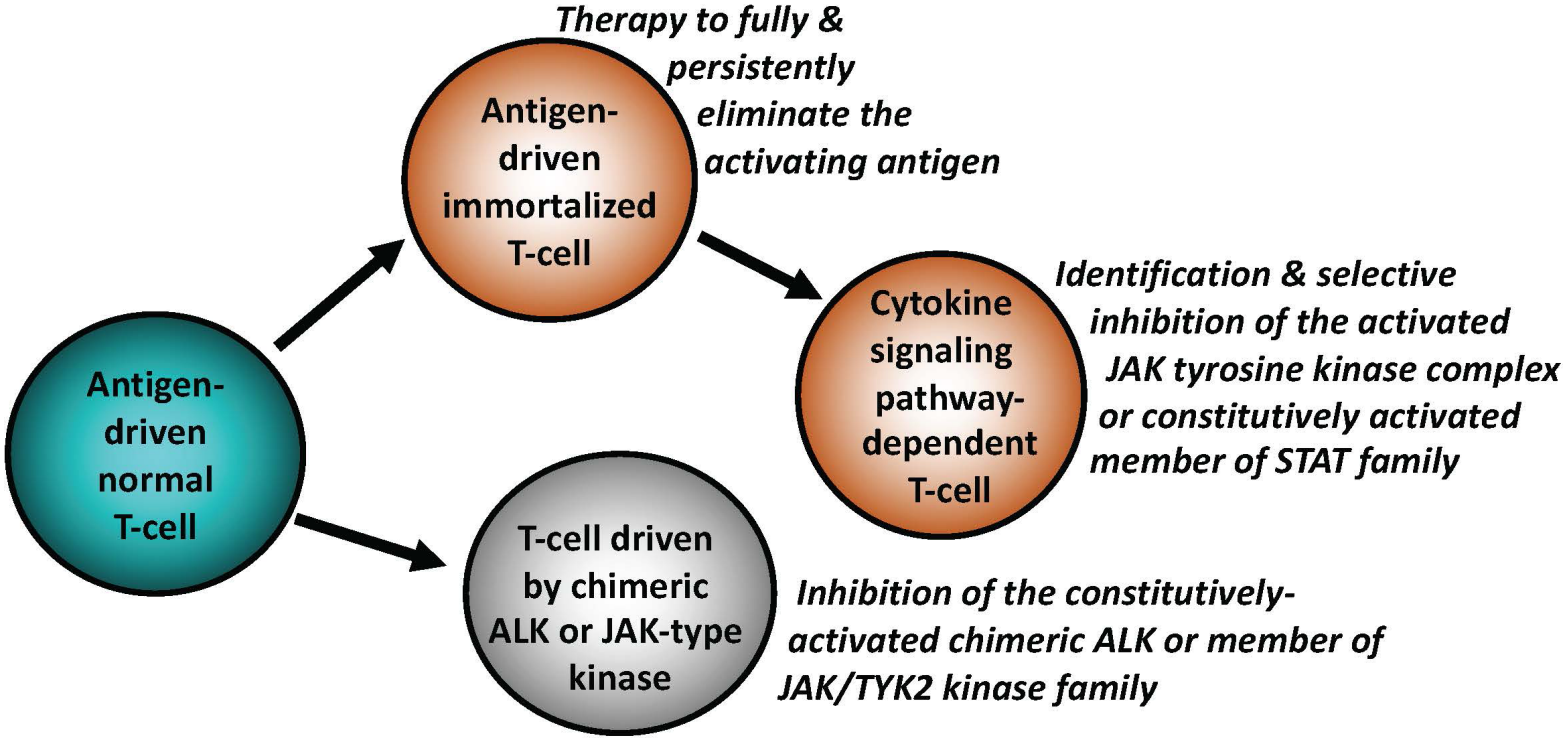
Key mechanisms of malignant cell transformation in T-cell lymphomas. Similar to indolent B-cell lymphomas, a subset of T-cell lymphomas remains antigen-dependent. The antigen-independence can be gained by T cells by activating signaling pathways downstream of cytokine receptors, physiologically required for activation and functional maturation of normal T cells. This occurs foremost through structural genetic alterations of the members of JAK and/or STAT families, resulting in their constitutive activation. Chimeric ALK (typically NPM1::ALK) is able to transform normal T cells by effectively substituting for the persistent JAK signaling. Similar to B-cell lymphomas, the types of targeted therapies may need to be adjusted to the specific T cell-transforming mechanisms, as listed.

As described in-depth elsewhere (1, 2), antigen-driven activation of the BCR and TCR with engagement of downstream signaling pathways is central for proper function, survival, development, and proliferation of normal B- and T-cells. Antigen dependence has been implicated in the pathogenesis of both B-cell (**Figure 1**) and T-cell lymphomas (**Figure 2**), particularly the ones from the indolent, low-grade B-cell lymphoma category. The *Helicobacter pylori (H. pylori)-*driven gastric mucosa-associated lymphoid tissue (MALT) B-cell lymphoma represents the prototypical example of an antigen-dependent lymphoma. Accordingly, therapeutic eradication of *H. pylori*, results in gastric MALT remission in the majority of patients.

The role of gluten in the development of celiac disease and the related enteropathy-associated T-cell lymphoma (EATL) of the small intestine is another example of antigen-dependent lymphoma. However, gluten elimination in EATL appears therapeutically less effective than *H. pylori* eradication in gastric MALT using antibiotics, possibly because ingestion of gluten in even trace amount may be sufficient to sustain survival of aberrant T cells. Accordingly, ype I of celiac disease considered to be refractory to gluten elimination from the diet (RCD) is characterized by a polyclonal proliferation of seemingly phenotypically and genetically normal T cells, while type II RCD represents a clonal expansion of phenotypically altered T-cell clones, with frequent activating mutations of JAK-STAT pathway (3). These striking differences suggest that the former may, indeed, be a form of aberrant, allergic-type immune response to traces of gluten and the latter may represent progression to a genuine low-grade EATL.

Other lymphomas, documented or postulated to be antigen-driven, include chronic lymphocytic leukemia/small lymphocytic lymphoma (CLL/SLL) responding to non-muscle myosin heavy chain IIA (4), LDL receptor-related protein-associated protein 1 (LRPAP1)-stimulated mantle cell lymphoma (MCL) (5), hepatitis C virus (HCV)-related splenic marginal zone lymphoma (SMZL), *Borrelia burgdorferi*-dependent primary cutaneous marginal zone lymphoma (PCMZL), *Chlamydia psittaci*-activated MALT of ocular adnexa, and *Moraxella catarrhalis*-promoted nodular lymphocyte-predominant Hodgkin lymphoma (NLPHL) (6). The evidence of ongoing immunoglobulin gene somatic hypermutations, preferential selection of specific MHC molecules, and formation of lymphoepithelial lesions in extra nodal, epithelia-associated lymphomas, all further indicate a pathogenic role of antigens in certain types of lymphoma.

In analogy to the curative effects of *H. pylori* elimination in gastric MALT, identification of the pathogenic antigen in a patient with a type of lymphoma in which antigen-dependence is highly likely, may pave the way for therapy aimed at the elimination, or at least blocking, as it may be the case with autoantigens, of such lymphomagenic antigen (**Figures 1**, **2**).

## BCR-dependent and -independent activation of BCR down-stream signaling pathways

BCR-mediated cell signaling in the absence of identifiable antigenic ligand gained most attention in the context of diffuse large B-cell lymphoma (DLBCL) (2). Chronic active BCR signaling is highly characteristic of the activated B-cell/non-germinal center (ABC/non-GC) subset of DLBCL. Accordingly, activating mutations of the BCR component CD79B (Y196H) and intracytoplasmic signaling molecule MYD88 (L265P) are a hallmark of the molecularly-defined MCD/C5 subtype within this DLBCL category. It is theorized that these mutations disrupt endocytic internalization of the BCR and permit spontaneous assembly of the signaling myddosome complex resulting in chronic activation of the down-stream signaling pathways, foremost NF-KB pathway. Of note, activating mutations of CARD11, a member of a key signaling complex downstream of the BCR, have also been identified in DLBCL, most likely leading to BCR independence. Accordingly, in mouse models mutated CARD 11 drives B cells into dysregulated plasmablastic differentiation via NF-KB and JNK activation, independently of BCR.

The presence of activating mutations of BCR and its down-stream signaling pathways, particularly the MYD88 mutation, have promising direct therapeutic implications. In early studies, patients with the MCD/(C5) subtype of DLBCL treated with the combination of standard R-CHOP immunochemotherapy and ibrutinib, a first-generation inhibitor of Bruton’s tyrosine kinase (BTK), the key signaling molecule down-stream of BCR, demonstrated long-lasting remissions (7).

It is generally underappreciated that the highly-transformed (high-grade) lymphomas such as DLBCL can undergo cell-signaling reprogramming due to additional mutations of BCR and members of its down-stream signaling pathways, including evidence of development of resistance to the previously applied targeted therapy (8). Consequently, molecular evaluation of DLBCL and other types of lymphoma, both at presentation, and at other clinical stages, particularly after post-therapy recurrence, may provide important insights into the mechanism(s) of lymphoma progression and reveal new therapeutic targets. The increasing accessibility to the extensive DNA and RNA sequencing using formalin-fixed paraffin-embedded tissues, allows for comparative analysis of serial samples from individual patients. This may further characterize the phenomenon of lymphoma reprogramming (9), and enable individualized therapy based on tumor-specific molecular status at a given disease stage (**Figure 1**).

The growing body of evidence shows that lymphoma cells are capable of becoming fully independent of BCR-mediated intracellular signaling and develop alternative cell-activation triggering mechanisms. Receptor tyrosine kinase-like orphan receptor 1 (ROR1), emerges as a prime example of this kind of lymphoma-cell reprogramming. ROR1, a cell-surface receptor with kinase structure, is normally expressed in the fetal liver and, likely, by cells in the parathyroid, pancreatic islets, adipose tissue, and gastrointestinal tract in adults (10). In hematopoietic cells, its expression is limited to early B-cell precursors (hematogones). Importantly, ROR1 expression has been identified in lymphoid malignancies derived from mature B lymphocytes including CLL/SLL, hairy cell leukemia (HCL), MCL, DLBCL, and follicular lymphoma (FL). ROR1 forms a complex with the CD19, a cell-surface receptor physiologically down-stream of the BCR signaling pathway. ROR1-CD19 complex activates key intracellular signaling pathways (10), including PI3K-AKT, MEK-ERK, and NF-kB (10, 11), effectively substituting for the BCR-induced cell signaling. Ectopic ROR1 expression has relevant translational implications as a target for immunotherapy using either ROR1 antibody-drug conjugate (12), ROR1-CD3 bispecific engager (13), or ROR1-targeting CAR T cells (14). In ongoing clinical trials with the anti-ROR1 CAR T-cells, no significant toxicities to normal tissues have been noted (10).

Furthermore, by bypassing BCR activation, ROR1 leads to BTK-inhibitor resistance in preclinical models of MCL (11). While the existence of this mechanism of resistance to BTK inhibition remains to be confirmed in lymphoma patients, monitoring ROR1 expression may become an important component of managing lymphomas treated with a BTK inhibitor. The need for ROR1 expression monitoring may be particularly applicable to MCL, given that mutations of BTK or its down-stream target PLCγ leading to BTK inhibitor resistance are extremely rare in MCL, in contrast to SLL/CL in which the mutations are highly prevalent (14). Overexpression of transcription factor EGR1 resulting in metabolic reprogramming to oxidative phosphorylation (OXPHOS) has also been recently implicated in BTK-inhibitor resistance in MCL and ABC subtype of DLBCL (15). Of note, malignant cells isolated from such lymphomas become sensitive to OXPHOS inhibitors, suggesting that targeting OXPHOS and EGR1, either separately or in combination, may prove efficacious in patients with lymphomas overexpressing EGR1.

In addition to ROR1, other aberrantly expressed cell-surface receptors may play a pathogenic role in B-cell lymphomas by at least partially substituting for BCR-generated cell signaling. For example, a functional interplay between mutated NOTCH1 and BCR has been identified in CLL/SLL and in ~1/3 of CLL/SLL cases which have undergone large-cell transformation (Richter transformation: RT) (16). This finding suggests that NOTCH1 plays a role in the disease progression and, consequently, may become a therapeutic target, particularly in RT, the clinically aggressive type of lymphoma with poor prognosis. *NOTCH1* mutations have been found also in ~1/3 cases of the clinically aggressive blastoid variant of MCL (17), further suggesting a link between NOTCH1 and lymphomas with high-grade features. Furthermore, increased expression of Toll-like receptors (TLR) including TLR9 as well as of WNT ligands, mostly WNT5a, has been identified in a subset of RT cases, in the context of decreased BCR expression (18). This suggests that TLR and WNT signaling compensate for diminished BCR signaling and, consequently, may become therapeutic targets. Finally, AXL, a cell-surface receptor with an intrinsic tyrosine kinase activity, has been implicated in BCR-independent cell signaling (19) and drug resistance (20) in CLL/SLL. Of note, a recent study (21) reports that constitutively activated AXL3 variant is expressed in MCL and its inhibition has proven therapeutically effective in preclinical *in vitro* and *in vivo* models.

## Dedifferentiation of B-cell lymphomas

Although rare, the development of DLBCL with phenotypic and genomic evidence of immaturity following the mature B-cell neoplasms: FL and DLBCL, has been now firmly recognized (22–25). These DLBCL/lymphoblastic B-cell lymphoma/leukemia (LBLL) “chimeras” typically are TdT+, CD10+, CD45^low^, and cell-surface Ig-negative, attesting to their at least partially immature phenotype. Strikingly all these chimeric TdT+ DLCBCL/LBLL cases reported to date acquired the *c-MYC* gene translocation, which indicates a critical role of the highly overexpressed *c-MYC* in this transformation. Translocations of *BCL-2* and/or *BCL-6* in both pre-and post-dedifferentiation stages are another common feature, which raises the possibility that defects in double-stranded DNA break repair contribute to the emergence of DLCBCL/LBLL. Accordingly, three cases of TdT+ DLBCL/LBLL demonstrated expression of *RAG1* (25), the gene encoding for an enzyme inducing DNA breaks to initiate immunoglobulin gene V(D)J recombination in immature B cells.

An in-depth molecular analysis of TdT+ DLBCL/LBLL cases has established a clonal relationship with preceding them FL and DBCL, by both shared rearrangement of immunoglobulin heavy chain (*IgH)* gene and identical mutations of other genes, with additional mutations seen at the DLBCL/LBLL stage (22–25). Of note, all TdT+ DLBCL/LBLL cases tested so far (22, 23) have displayed somatic hypermutations of the *IgH* gene, indicating that these lymphomas have indeed dedifferentiated from mature lymphoma cells, rather than have originated from a common, immature malignant progenitor B cell. This unique entity of DLBCL/LBLL poses substantial diagnostic and clinical challenges, as it has a phenotype to large degree mimicking acute B-cell lymphoblastic leukemia (B-ALL) and responds poorly to diverse therapy protocols, including those used for B-ALL.

## Transdifferentiation of B-cell lymphomas

Transdifferentiation is a striking example of lineage plasticity in malignant cells. Although this phenomenon was originally identified over two decades ago with the description of FL turning into histiocytic/dendritic cell neoplasms (HDCN) (26), it attracted recently much more attention, in the era of targeted kinase-inhibitor and chimeric antigen receptor (CAR) T-cell-based immunotherapy (27). While initially recognized solely by *IgH* gene rearrangements shared between the preceding FL and ensuing HDCN, recent advances in DNA and RNA sequencing have enabled the identification of clonal relationship between lymphoma and co-occurring or following HDCN, based also on shared profiles of gene translocations and mutations of *non-IgH* genes (27).

There are currently well-documented cases of HDCN transdifferentiating from not only FL but also CLL/SLL, MCL, MZL, DLBCL (27), and Burkitt lymphoma (BL) (28) with transdifferentiation from FL and CLL/CLL appearing to be the most common. The substantial genomic mutational heterogeneity in these ‘secondary’ HDCN’s likely stems at least in part from the high diversity among the underlying lymphomas. However, in spite of their mutational heterogeneity, comprehensive DNA sequencing studies of these HDCN revealed frequent mutations in members of the MAPK pathway, including *KRAS* (6/16 cases examined), *BRAF* (4/16), and *MAPK2K1* (3/16). This raises the possibility that MAPK pathway components may become novel therapeutic targets in a large subset of the transdifferentiated HDCN (27).

In addition to morphing into HDCN, transdifferentiation of MCL to poorly differentiated sarcoma with evidence of neuromuscular differentiation has been reported (29, 30), which demonstrates even more striking lineage plasticity potential of malignant lymphocytes. Genome-scale shift in DNA methylation was identified in the sarcoma vs. the preceding MCL cells with >12,000 gene promoters profoundly reprogramming their methylation pattern (29). This sweeping change was associated with silencing of genes involved in B-cell differentiation and partial activation of differentiation programs towards neural and muscle cells (29). While the underlying mechanisms of this profound cell reprograming remain unclear, mutations of retinoblastoma 1 (*RB1)* and *TP53* genes were identified in these transdifferentiated sarcoma cases (29, 30). It is quite intriguing in this context that mutations of these two genes were also detected in poorly differentiated neural tumors transdifferentiated from carcinomas of lung (31) and prostate (32), occurring in response to EGFR inhibition and anti-androgen signaling therapy, respectively. Therefore, it could be surmised that the *RB1* and *TP53* mutations may play a key role in transdifferentiation of lymphoid and non-lymphoid neoplasms towards neural cell lineage.

Upon transitioning from MCL to sarcoma, the transdifferentiated sarcoma cells gained expression of several non-lymphoid cell receptors and displayed sensitivity to pharmacologic inhibition of one of these receptors, namely FGFR (29). This finding combined with the epigenetics-driven cell reprogramming indicates emergence of newly acquired cell-growth dependency of the transdifferentiated cells. It could be potentially exploited therapeutically in the patient setting by targeting DNA methylation machinery and the new oncogenic drivers such as FGFR. Of note, FGFR1 expression has been recently identified also in advanced MCL, including BTK inhibitor-resistant cases (33). Furthermore, FGFR1 inhibition has shown efficacy in pre-clinical *in vitro* and *in vivo* MCL models (33), suggesting that FGFR1 may become a therapeutic target in this type of lymphoma, even without evidence of overt transdifferentiation.

The triggering events leading to transdifferentiation remain mostly unclear. While the lineage change may be spontaneous, therapeutic pressures are more likely to underlie, or accelerate, the process. In analogy to the above-mentioned carcinomas of the lung and prostate transdifferentiating in response to therapies targeting EGFR (31) and androgen (32) signaling, respectively, targeted therapies may be responsible for lymphoma reprogramming from the dependence on BCR signaling. Prolonged exposure to kinase inhibitors such as BTK inhibitors as seen in the MCL case morphed into sarcoma (29), could certainly be a factor promoting BCR/BTK independence including transdifferentiation and a related profound cell-signaling reprograming, as a very drastic mechanism of drug resistance.

CAR T-cell therapy targeting CD19 (CART19) may also play a role in lymphoma reprograming including transdifferentiation by enabling outgrowth of the preexisting non-lymphoid subclone, due to the CART19-mediated eradication of the dominant lymphoma clone expressing CD19 (29). Of interest, comparative analysis of pre- and post-CART19 therapy tissue samples collected from nine patients with high-grade B-cell lymphomas who failed this immunotherapy, revealed not only at least a partial decrease in CD19 expression by eight of the lymphomas but also less frequently seen losses of other B-cell markers CD79A, CD20, and PAX5 (34). These findings suggest a genuine loss of mature B-cell identity in some of the cases, likely promoted by CART19-mediated selection of the preexisting aberrant subclones with at least partial reprogramming away from the B-cell lineage. Accordingly, histiocytic differentiation of a subset of the malignant B cells was noted upon relapse in one case and T-cell differentiation in another one (34), indicating that CART19 therapy can indeed occasionally enable outgrowth of cells displaying transdifferentiation towards a non-B-cell lineage. Finally, the relapsed tumors acquired a number of mutations in genes not typically altered in B-cell lymphomas such as *KRAS, MAP2K2, PIK3R1, and PIK3R2*, further supporting their loss of B-cell identity and suggesting that targeting PI3K and KRAS in such cases may prove therapeutically effective.

## Malignant transformation of T and B lymphocytes driven by chimeric anaplastic lymphoma kinase

While the establishment of an overt lymphoid malignancy is almost universally a stepwise process, lymphomas expressing anaplastic lymphoma kinase (ALK) and, seemingly, similarly powerful oncogenic kinases (35–37) are a notable exception (**Figure 2**). ALK, typically expressed as the NPM1-ALK (NPM1::ALK) hybrid protein due to t(2;5) chromosomal translocation resulting in fusion of the involved genes, profoundly reprograms targeted CD4+ T cells by conferring upon these cells features required for a full-fledged malignant cell transformation (38, 39). These NPM1::ALK-imposed oncogenic features are achieved to a large degree through activation of cell signaling pathways triggered in normal T cells by cytokines, foremost activation of transcriptional regulator STAT3 (35). These oncogenic properties include cell immortalization and protection from cell death, enabled at least in part by activation of stem cell-related genes (40), induction of multi-faceted inhibition of anti-tumor immune response (35), epigenetic silencing of TCR- and cytokine-signaling genes acting in the context of NPM1::ALK as tumor suppressors (35), protection from hypoxia (35), and mobilization of the metabolic NAD synthesis salvage pathway to sustain enzymatic activity of the oncogenic ALK (41). Given the above diverse pro-oncogenic effects of NPM1::ALK, it is not surprising that targeting ALK clinically induces complete, long-lasting remissions in response to crizotinib, the first generation ALK inhibitor (42, 43). However, resistance to ALK-inhibition monotherapy has emerged (44), even to the second- and third-generation, highly potent ALK inhibitors, providing rationale for a combination therapy to prevent development of the resistance. ALK inhibition already has, or could be, combined with either standard chemotherapy (45), CD30 antibody-toxin conjugate (46), or, preferably, agents targeting pathways playing a role in ALK-driven lymphomas (35). Similar to NPM1::ALK, another form of chimeric ALK involving clathrin (CLTC::ALK) which is expressed in a distinct subtype of B-cell lymphoma displaying plasmablastic cell morphology and immunophenotype, is also highly responsive to the next generation ALK inhibitors (47), further highlighting the potent oncogenic role of chimeric ALK, regardless of the T- vs. B-cell lineage of the transformed lymphocytes (**Figures 1**, **2**).

## Malignant transformation of T lymphocytes driven by other potent oncogenic kinases

ALK-negative anaplastic large cell lymphomas (ALCL) are clinically and molecularly quite heterogeneous. Based on their presentation, they are classified as systemic, primary cutaneous, and breast implant-associated (48). Despite their heterogeneity, common pathogenic mechanisms have emerged. Accordingly, rearrangements of chromosome 6p25.3 involving *DUSP22* and *IRF4* gene region characterize distinct subset of both cutaneous and systemic ALK-negative ALCL lacking STAT3 activation (48, 49). These chromosome 6p25.3 rearrangements confer an overall better prognosis, according to some studies (48, 49). In other ALK-negative ALCL cases, translocations and related persistent expression of genes encoding tyrosine kinases, to some degree functionally analogous to ALK, has been identified (36, 37, 48). While one of these ALK-like kinases ROS1, is structurally closely related to ALK, the other two:TYK2 and JAK2 are members of the JAK tyrosine kinase family. They all activate similar signaling pathways, foremost STAT3, considered the key transcriptional effector for these chimeric oncogenic kinases (37). Of note, systemic and cutaneous ALCL lacking any of these chromosomal translocations and the related constitutively active fusion kinases, frequently display activating point mutations of *JAK1*, *JAK3*, and/or *STAT3* (37). Similar point mutations of these genes occur also in breast implant-associated ALCL (48). These findings further point towards the importance of the signaling pathways physiologically activated in normal T cells by certain cytokines for the entire category of ALCL.

Mutation-driven aberrant activation of selected JAKs and STATs has also emerged as a common denominator for T-cell lymphomas beyond ALCL. For example, in addition to the pathognomonic alterations of the TCL1 gene, T-cell prolymphocytic leukemia (T-PLL) frequently displays point mutations of *JAK1* (6.3% of cases), *JAK3* (36.4%), and *STAT5B* (18.8%) (50). Furthermore, ~70% of cases show losses of negative regulators of JAKs and STATs including SHP1, SOCS1, and SOCS3 (50), further stressing pathogenic role of constitutively active JAK and STAT signaling in T-PLL. A large subset (40%) of NK-cell large granular lymphocytic leukemia (LGL) harbors mutations of *STAT3* gene, the same percentage of T-cell LGL (T-LGL) cases shows mutations of *STAT3* and *STAT5B* genes (51). Mutations of these two oncogenes are also frequently seen in NK and γδ T-cell lymphomas (37). In turn, cytotoxic CD8+ T-cell lymphomas of skin, particularly the primary cutaneous, aggressive and epidermotropic cytotoxic T-cell lymphoma (PCAECyTCL) show alterations of predominantly *JAK2* but also *JAK3*, *STAT5B, and SOCS1* genes, among other genomic changes (52, 53). Similarly, a subset of monomorphic epitheliotropic intestinal T-cell lymphoma (MEITL) was found to frequently carry mutations in *STAT5B* (57%), *JAK3* (50%), and *JAK1* (12.5%). in addition to deletion or expression loss of the *SETD2* gene (90%) (54), encoding the histone H3 lysine 36 histone (H3K36) methyltransferase with tumor suppressor gene features. Finally, treatment with ruxolitinib, the kinase inhibitor preferentially impairing activity JAK1 and JAK2, showed therapeutic efficacy against diverse T-cell lymphomas, particularly the ones with evidence of *JAK*/*STAT* mutations or even STAT3 activation, foremost in T-LGL (55). These clinical results further indicate oncogenic role of the above-listed JAKs and STATs in the T-cell malignancies and support the notion that the oncogenic forms of JAK family members emerge as attractive therapeutic targets in the whole spectrum of mature T-cell lymphomas and leukemias (**Figure 2**). Mutated and, hence, constitutively activated members of the STAT family such as STAT3 and STAT5B could also become important therapeutic targets with a number of their direct inhibitors already developed. However, clinical efficacy of these inhibitors still remains to be convincingly proven.

## Epstein-Barr virus as substitute of BCR signaling

EBV-driven lymphoproliferative disorders, mostly of B-cell origin (56), are another example of a seemingly one-step oncogenesis (**Figure 1**) in at least some cases with a number of the EBV-encoded genes playing a role in the malignant cell transformation (57). The dominant EBV-encoded oncogenes belong to the late membrane protein (LMP) and Epstein-Barr nuclear antigen (EBNA) families; the former is largely responsible for the considerable immunogenicity of the EBV-dependent lymphoproliferations. This strong immunogenicity limits the occurrence of these disorders to immune-compromised individuals, unless the immunogenicity is greatly diminished. As seen in BL, this can occur via gene translocation-triggered constitutive activation of MYC and mutation of other oncogenes, permitting down-regulation of the presumably highly immunogenic EBV-encoded oncogenic drivers such as LMP1 and LMP2. EBV succeeds in transforming target cells to a large degree by engaging signaling pathways, normally activated by BCR (58) and, hence, by effectively hijacking the physiological mechanisms of B-cell activation. Recent study (59) identified in EBV-positive DLBCL relatively frequent mutations of *SOCS1*, *NOTCH2*, and *NOTCH1* genes, suggesting that these mutated genes may have a pathogenic role in this type of lymphoid malignancy. While current therapies for in transplant patients affected by the EBV-driven lymphoproliferative disorders typically include tempering immunosuppressive regimens, often combined with CD20-targeting immunotherapy, or multiagent immunochemotherapy (60), the conceptually most advanced clinical trials focus on immunotherapy with EBV-detecting cytotoxic T cells (61) LMP-targeting CAR T cells (62), or anti-EBV vaccines (62).

## Conclusions

In summary, in-depth molecular analysis of lymphomas including relatively limited longitudinal studies of tissue samples from consecutive biopsies obtained from the same patients, has revealed remarkable plasticity of malignant lymphoid cells. Lymphomas employ the entire spectrum of signaling mechanisms for survival and growth, spanning from antigen dependence to antigen receptor-dependence in an antigen-independent manner, to reliance on signaling pathways activated independently of BCR (**Figure 1**) or TCR (**Figure 2**). While the advanced B-cell lymphomas often depend on mutations of BCR or members of its down-stream signaling pathways, T-cell lymphomas tend to develop activating mutations of selected JAKs and STATs, normally activated by cytokines critical for T-cell development and function. Furthermore, malignant lymphocytes are capable of profound cell-signaling rewiring leading in the most extreme cases to transdifferentiation, i.e. loss of their original lymphoid lineage identity and at least partial acquisition of differentiation towards a different, non-lymphoid mesodermal lineage. The frequency of lymphoma reprogramming is expected to grow in the era of targeted therapies, particularly in monotherapy targeting a member of BCR-dependent cell signaling pathways. An in-depth understanding of these diverse and sometimes complex cell-activating mechanisms including identification of related biomarkers is required for the development of effective and highly individualized therapeutic interventions based not only on the pathogenic features of a given lymphoma at clinical presentation but also at its recurrence.

## Author contributions

MW: Conceptualization, Writing – original draft, Writing – review & editing. PK: Writing – original draft, Writing – review & editing. RN: Investigation, Writing – original draft.
